# PW01-040 – Definition of polymorphism C3435T MDR1 gene in JIA

**DOI:** 10.1186/1546-0096-11-S1-A93

**Published:** 2013-11-08

**Authors:** F Rokhlina, G Novik

**Affiliations:** 1 SPbGPMU, St. Petersburg, Russian Federation

## Introduction

Juvenile idiopathic arthritis (JIA) is one of the most common rheumatic disease in children.

Brug of choice is methotrexate (MTX) - cytostatic drug from the group of antimetabolites, folic acid antagonists. According to various authors, about 70% of patients receiving therapy with MTX are in remission for the disease.

In 1988, MTX was approved FDA (Food and Drug Administration, USA) for the treatment of rheumatoid arthritis. Currently, MTX is the main drug in the treatment of JIA. Preferred therapeutic recommendations on the number of MTX based on a randomized, double-blind, placebo-controlled study conducted in 1992.

MDR1-gene product - the P-glycoprotein (P-gp), the protein acts as a trans-membrane pump, and affects the activity of many drugs. Polymorphism in the gene MDR1, may affect the pharmacokinetics of many drugs, including anticancer drugs.

According to the authors of Jinwei Chen (2011) in adult patients with rheumatoid arthritis (RA) in the Chinese population C3435T MDR1 gene polymorphism may be associated with susceptibility to RA, but may influence the effectiveness of antirheumatic therapy of RA, and CC genotype may be associated with immune rheumatoid arthritis (RRA).

## Objectives

The aim of our study is to determine the effect on the efficiency of the MDR1 gene therapy JIA.

## Methods

Were examined 103 patients diagnosed with juvenile idiopathic arthritis (according to the EULAR), receiving standard treatment with methotrexate at a dose of 15 mg/m2 over 3 months (intramuscular). All children were defined P-glycoprotein (the product of the gene MDR1), and C3435T polymorphism of the gene MDR1. A genetic study was carried out using polymerase chain reaction followed by restriction analysis to determine the C3435T polymorphism of the gene MDR1. Modeling inflammation in vitro was carried out by treating the blood with recombinant human interleukin 2, followed by determination of the level of P-glycoprotein (CD243-PE) on peripheral blood lymphocytes with monoclonal antibodies by flow cytofluorometry.

## Results

Among the 103 children included in the study - 65 (63.11%) females and 38 (36.89%) boys with different forms of juvenile idiopathic arthritis: polyarthritis - 46 children (44.66%), oligoarthritis - 25 children (24, 27%), systemic arthritis - 14 children (13.59%), arthritis entezitassociated - 18 patients (17.48%).

Analysis of changes in the level of P-glycoprotein after IL2 stimulation, depending on the genotype of MDR1 gene showed significant differences. When comparing the patients in these groups, it was found that in the group of children with genotype TT, increase P-IL2 protein after stimulation were significantly lower than in children with genotype CT. In patients with genotype TT average sedimentation rate was significantly higher than that of children in the group CT in the acute phase, and in the waning activity of the inflammatory process in the joints. Similar changes were obtained by comparing the genotypes CC + CT and TT.

## Conclusion

Given the data, we can assume that children with the TT genotype MDR1 gene (C3435T) is worse respond to standard treatment of JIA.

## Disclosure of interest

None declared

